# Theoretical Analysis of InGaAs/InAlAs Single-Photon Avalanche Photodiodes

**DOI:** 10.1186/s11671-018-2827-4

**Published:** 2019-01-03

**Authors:** Siyu Cao, Yue Zhao, Shuai Feng, Yuhua Zuo, Lichun Zhang, Buwen Cheng, Chuanbo Li

**Affiliations:** 1 School of Science, Minzu University of China, Beijing, 100081 China; 2 State Key Laboratory on Integrated Optoelectronics, Institute of Semiconductors, Chinese Academy of Sciences, Beijing, 100083 China; 3 Center of Materials Science and Opto-Electronic Engineering, University of Chinese Academy of Sciences, Beijing, 100049 China; 4 School of Physics and Optoelectronic Engineering, Ludong University, Yantai, 264025 China

**Keywords:** Single-photon avalanche photodiodes, Theoretical analysis, Simulation, Tunneling effect

## Abstract

Theoretical analysis and two-dimensional simulation of InGaAs/InAlAs avalanche photodiodes (APDs) and single-photon APDs (SPADs) are reported. The electric-field distribution and tunneling effect of InGaAs/InAlAs APDs and SPADs are studied. When the InGaAs/InAlAs SPADs are operated under the Geiger mode, the electric field increases linearly in the absorption layer and deviate down from its linear relations in the multiplication layer. Considering the tunneling threshold electric field in multiplication layer, the thickness of the multiplication layer should be larger than 300 nm. Moreover, SPADs can work under a large bias voltage to avoid tunneling in absorption layer with high doping concentrations in the charge layer.

## Background

In_0.53_Ga_0.47_As/In_0.52_Al_0.48_As (hereafter referred to as InGaAs/InAlAs) and InGaAs/InP avalanche photodiodes (APDs) are the most significant photodetectors for short-wave infrared detection. In recent years, research on quantum key distribution has quickly progressed, and now InGaAs/InAlAs and InGaAs/InP APDs can realize the single-photon counting and timing as single-photon APDs (SPADs) [1]. Compared with other single-photon detectors in the SWIR wavelength range, such as photomultiplier tubes, InGaAs single-photon avalanche diodes have the distinctive advantages of high performance, high reliability, low bias, small size, good time resolution, and ease of operation [2, 3]. Thus, InGaAs/InAlAs and InGaAs/InP APDs are attracting the considerable attentions [4, 5]. Compared with APDs operating in linear mode, APDs operated in Geiger mode as SPADs are applied with a reverse bias that exceeds the breakdown voltage [6]. SPADs achieve a high gain in the multiplication layer, and a single photon can trigger a macroscopic current pulse, which provides the ability to accurately sense the arrival at the detector of a single photon [7]. Thus, SPADs can detect the single photon at a wavelength of 1550 nm [8]. Meanwhile, the absorption wavelength can be controlled by the materials of absorption layer [9].

Compared with InAlAs-based SPADs, theoretical and simulation studies of InP-based SPADs are more comprehensive [2, 10–12]. However, InAlAs-based APDs are increasingly being used in place of InP-based APDs as they can improve performance both in APDs and SPADs [13]. The ionization coefficient ratio of electron (α) to hole (β) in InAlAs is larger than that in InP, thereby resulting in a low excess noise factor and high gain-bandwidth product in InAlAs-based APDs [14]. The larger band gap of InAlAs can improve the breakdown characteristics and decrease the dark count rate (DCR) in SPADs [15]. InAlAs-based APDs have high-electron mobility, leads to faster response times than that of InP-based APDs [16]. Moreover, ionization coefficient ratio of InAlAs APDs is less sensitive to temperature changes of InP-based APDs [17]. Consequently, InGaAs/InAlAs APDs can achieve high performance in terms of breakdown characteristics, DCRs, excess noise, gain-bandwidth, response time, and temperature characteristics.

Studies on InGaAs/InAlAs APDs have mainly focused on increasing the single-photon detection efficiency (SPDE) and decreasing the DCR in SPADs. Karve et al. demonstrated the first InGaAs/InAlAs SAPDs, which has a SPDE of 16% at 130 K [18]. Nakata et al. improved the temperature performance of SPADs, which achieves a SPDE of 10% at 213 K [19]. Zhao et al. designed a self-quenching and self-recovering InGaAs /InAlAs SPAD with a SPDE of 11.5% at 160 K; concurrently, a DCR of 3.3 M Hz has been observed [20]. Meng et al. designed a mesa structure InGaAs/InAlAs SPAD, which achieves a SPDE of 21% at 260 K [21]. Then, they applied a thick absorption and multiplication layer in a similar structure, which improves the SPDE to 26% at 210 K and decreases the DCR to 1 × 10^8^ Hz [22]. However, in these studies, the DCRs of InGaAs/InAlAs SPADs are too high compared with InGaAs/InP SPADs (in recent InP SPADs, DCRs are typical < 10^4^ Hz) [23]. The high DCRs in InGaAs/InAlAs SPADs are attributed to tunneling currents, which is caused by the high field at the over bias voltage [21, 22, 24]. Thus, decreasing tunneling-related mechanisms is significant for InGaAs/InAlAs SPADs, and these mechanisms are related to the electric-field distribution in SAPDs. From literatures [1. 9], the tunneling threshold electric field is 2.0 × 10^5^ V/cm in the absorption layer (InGaAs) and 6.8 × 10^5^ V/cm in the multiplication layer (InAlAs). Thus, a suitable electric-field distribution is significant for InAlAs SPADs, which is determined by the charge-layer and multiplication-layer thickness. Considering the charge layer of InAlAs APDs, Kleinow et al. studied the influence of doping concentration in this layer and found that doping concentration is more important for the performance of InGaAs/InAlAs APDs [25, 26]. Chen et al. studied the influence of the charge and multiplication layers on punch-through and breakdown voltages by theoretical analysis and simulation [27]. These studies have focused on the performance of InAlAs APDs under the linear model. However, the performance of InAlAs SPADs has not yet been fully understood under the Geiger mode.

In this paper, theoretical analysis and simulation are used to study the tunneling effect and electric-field distribution in InGaAs/InAlAs SPADs. With the consideration of tunneling threshold electric field under the Geiger mode, the design criteria of SPADs are optimized to avoid the tunneling effect.

## Methods

**Fig. 1:**
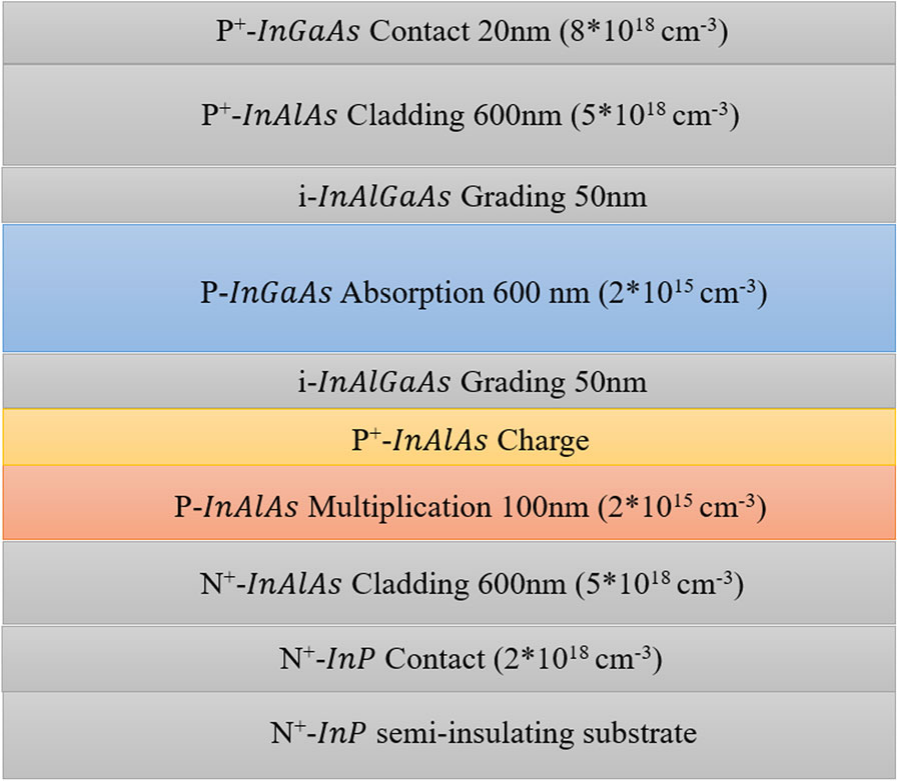
Schematic cross-section of the front-illuminated SAGCM APDs. Presents the schematic cross-section of the top-illuminated SAGCM InGaAs/InAlAs APD. It includes structure, materials, doping, and thickness. From bottom to top, the layers are sequentially named as substrate, contact layer, cladding layer, multiplication layer, charge layer, grading layer, absorption layer, grading layer, cladding layer, and contact layer

Numerical simulations are performed for the front-illuminated SAGCM InGaAs/InAlAs APDs by using TCAD [28]. The physical models used for simulation are presented as follows. The Selberherr impact ionization model simulates the avalanche multiplication in InAlAs. Electric-field distribution and diffusion current are described by the drift-diffusion model, which includes the Poisson and carrier continuity equations. Band-to-band and trap-assisted tunneling models are used for the tunneling current. Other basic models, including the Fermi–Dirac carrier statistics, Auger recombination, carrier-concentration dependence, Shockley–Read–Hall recombination, low field mobility, velocity saturation, impact ionization, and ray-tracing method are used in the simulation. The schematic cross-section of the front-illuminated APD epitaxial structure for the simulation is shown in Fig. 1.

From bottom to top, the layers are sequentially named as substrate, contact layer, cladding layer, multiplication layer, charge layer, grading layer, absorption layer, grading layer, cladding layer, and contact layer. The photogenerated carriers induced in the absorption layer drifts to the multiplication layer, where it triggers avalanche breakdown. The electric field in the absorption is adjusted using the charge layer control and maintain a high field only in the multiplication layer. Between the charge and absorption layers, an InAlGaAs grading layer avoids the electron pile-up at the InGaAs-InAlAs heterojunction. The device structure in our simulation is similar to the experimental structure in ref. [21].

The electric-field distribution in SAGCM APD can be solved with the Poisson equation, PN depletion-layer model, and boundary condition equation [29]. The Poisson equation is given as

1 $$ \frac{d\xi}{d x}=\frac{\rho }{\varepsilon }=\frac{q\ast N}{\varepsilon }. $$

The boundary condition equation is given as

2 $$ Vbias+ Vbi=-{\int}_0^w\xi \left(x,\mathrm{w}\right) dx. $$

In these equations, *ρ* is equal to the dopant ion *q* × *N* in the depletion-layer, *ε* is the dielectric constant of the material, *V*_*bias*_ is the bias voltage on the APDs, *V*_*bi*_ is the built-in potential, and *w* is the depletion-layer thickness. The mathematical relationship between electric-field distribution and bias voltage when the boundary of the depletion layer reaches the contact layer in the device can be derived using Eqs. (1) and (2).

The tunneling currents are composed of band-to-band and trap-assisted tunneling. Band-to-band tunneling current depends on the field in the material and becomes a dominant component of dark current at high fields [24, 30]. The generation rate of band-to-band tunnel is given as [31].

3 $$ {G}_{\mathrm{btb}}={\left(\frac{2{m}^{\ast }}{E_g}\right)}^{1/2}\frac{q^2E}{{\left(2\pi \right)}^3\mathrm{\hslash}}\exp \left(\frac{-\pi }{4q\mathrm{\hslash}E}{\left(2{m}^{\ast}\ast {E}_g^3\right)}^{\raisebox{1ex}{$1$}\!\left/ \!\raisebox{-1ex}{$2$}\right.}\right) $$

In the above equation, *E*_*g*_ is the energy band gap of InGaAs (0.75 eV) or InAlAs (1.46 eV), m* (equal to 0.04 *m*_*e*_ in InGaAs and 0.07 *m*_*e*_ in InAlAs) is the effective reduced mass, and *E* is the maximum electric field. *G*_btb_ depends on the electric field *E* and energy band gap *E*_*g*_, *w*_tunnel_ is assumed to be the effective thickness for the tunneling process, and *A* is assumed to be the area of the device. Thus, the tunneling current of the band-to-band tunnel is given as [13].

4 $$ {I}_{\mathrm{tunnel}}/A={G}_{\mathrm{btb}}\ast q\ast {w}_{\mathrm{tunnel}} $$

**Fig. 2:**
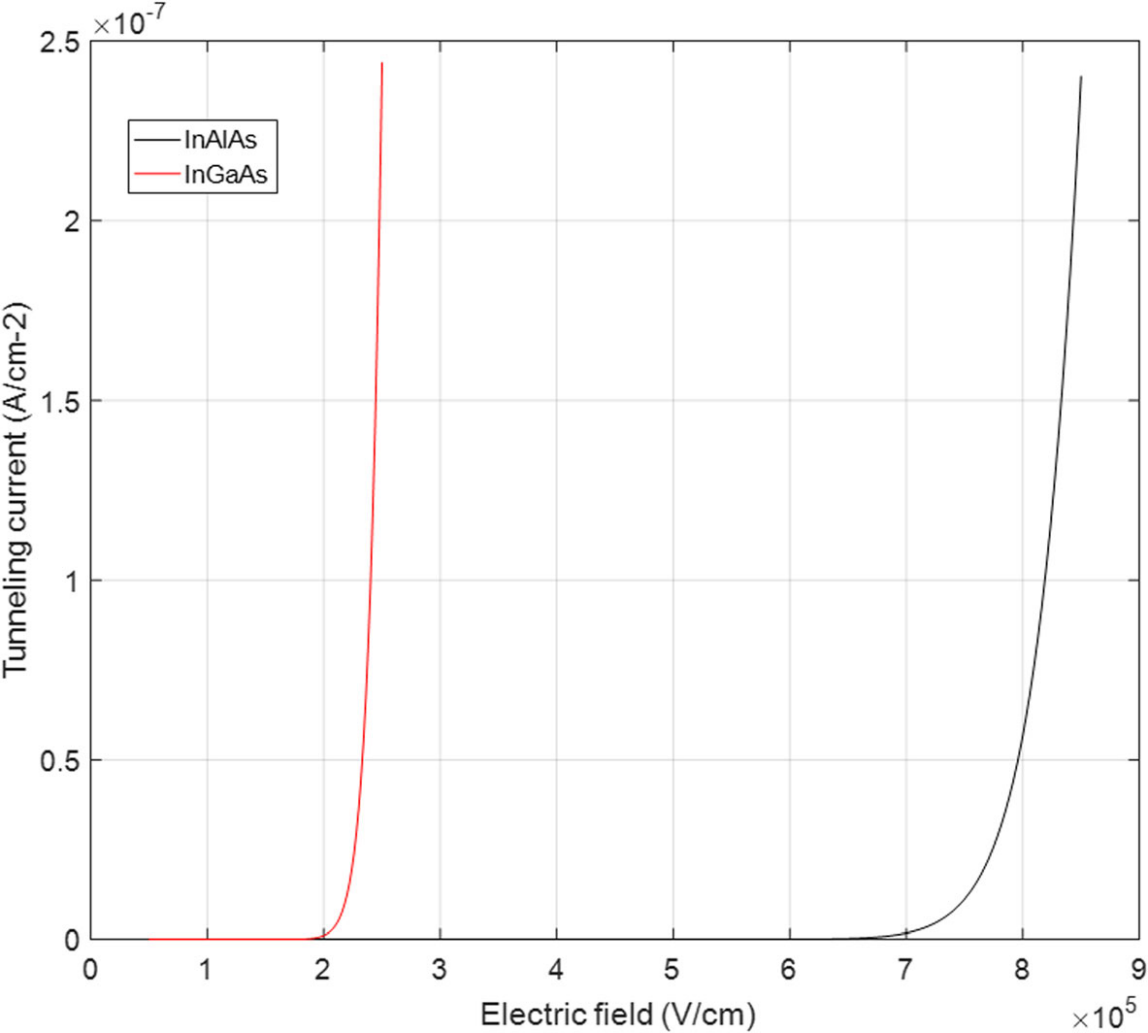
Relationship between *I*_tunnel_/*A* and electric field in InGaAs and InAlAs. Presents the calculated results of *I*_tunnel_/*A*. *I*_tunnel_ becomes significant at 2.0 × 10^5^ V/cm of InGaAs and 6.9 × 10^5^ V/cm of InAlAs, respectively

The calculated results of *I*_tunnel_ /*A* (*w*_tunnel_ = 1 μm) are presented in Fig. 2. *I*_tunnel_ becomes significant at 2.0 × 10^5^ V/cm of InGaAs and 6.9 × 10^5^ V/cm of InAlAs, respectively. We find that these calculated values correspond well with the tunneling threshold electric field (2.0 × 10^5^ V/cm, InGaAs) and (6.8 × 10^5^ V/cm, InAlAs) in references. The tunneling current can sufficiently influence the performance of SPADs at a high field. Thus, the field should be adjusted to lower than the tunneling threshold value both in the InGaAs and InAlAs of SPADs. Table 1 shows the parameters used in the simulation.

**Table 1:** Material parameters used in the simulation of InGaAs/InAlAs SAGCM APDs [33]

Parameter	Units	Electron	Hole
Energy band gap (InGaAs)	eV	0.75	
Energy band gap (InAlAs)	eV	1.46	
Impact coefficient a (InAlAs)	cm^−1^	2.1*10^6^	2.4*10^6^
Impact coefficient b (InAlAs)	V/cm	1.62*10^6^	1.86*10^6^
Effective threshold energy (InAlAs)	eV	3.2	3.5
SRH lifetime (InAlAs)	s	1*10^−6^	1*10^−6^

## Results and Discussion

**Fig. 3:**
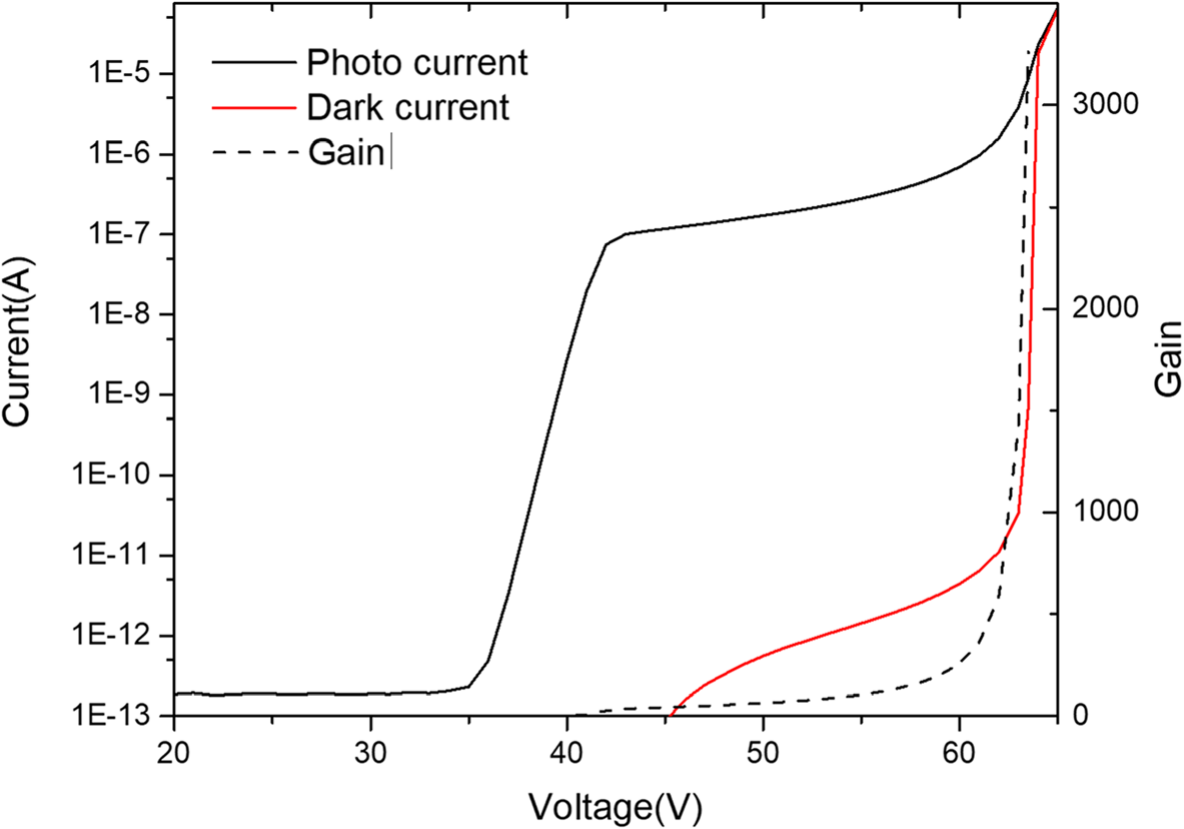
Current-voltage curve along with gain vs voltage of InGaAs/InAlAs APD. Presents the i-v curve along with gain vs voltage curve for some typical device structure as figure

In this section, the theoretical analysis and conclusions were studied by simulation. First, the electric-field distribution under Geiger mode was studied in section A. Then, with the consideration of tunneling threshold electric field under the Geiger mode, the design criteria of SPADs are optimized to avoid the tunneling effect in section B. The typical device structure in the reference [22] was used to test the simulation model. In this simulation, we used the same simulation engine as the reference [28] and the Current-voltage curve along with gain vs voltage curve were given by Fig. 3. It can be found that gain gradually increase after the punch-through voltage and sudden increase at breakdown voltage.

### Electric-Field Distribution Under the Geiger Mode

We found that the device performance is greatly influenced by the electric field distribution. To maintain the high gain and small dark current, the proper control of the electrical field in the multiplication and absorption layers is important. From the ref. [32], a suitable field distribution in InGaAs/InAlAs APD should comply with those rules. The guarantee V_pt_ (punch-through voltage) < V_br_ (breakdown voltage) and V_br_-V_pt_ should have a safety margin for processing variations in temperature fluctuations and operation range. At breakdown voltage, the multiplication gain goes toward infinity and the current sudden increase [32]. When the dark or photo current reached 50 μA, the corresponding voltage is called breakdown voltage V_br_. In the absorption layer, the electric field should be larger than 50–100 kV/cm to ensure enough velocity for the photo-induced carriers. Concurrently, the electric field must be less than 180 kV/cm to avoid the tunneling effect in the absorption layer. Electric field distribution greatly influences the device performance. The choice of electric field in the absorption layer has a balancing of the trade-off between small transit time, dark current, and high responsivity for the practical requirement.

**Fig. 4:**
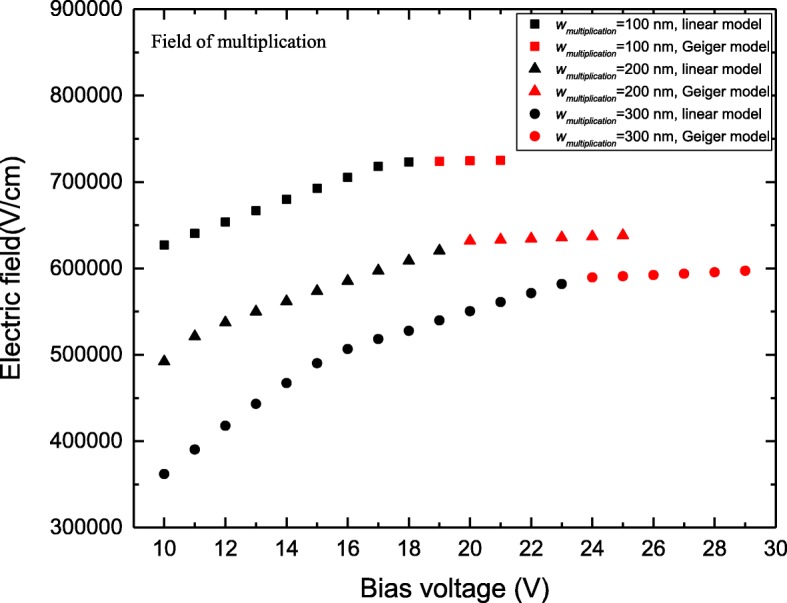
Simulation results electric field in multiplication under the Geiger mode. The values of *W*_multiplication_ is 100 nm (black square), 200 nm (black triangle), 300 nm (black circle). Figure 3 presents the simulated field-voltage characteristics in the multiplication layers under the Geiger mode. The thickness of the charge layer is 50 nm, and the thicknesses of the multiplication layer are 100, 200, and 300 nm, respectively

**Fig. 5:**
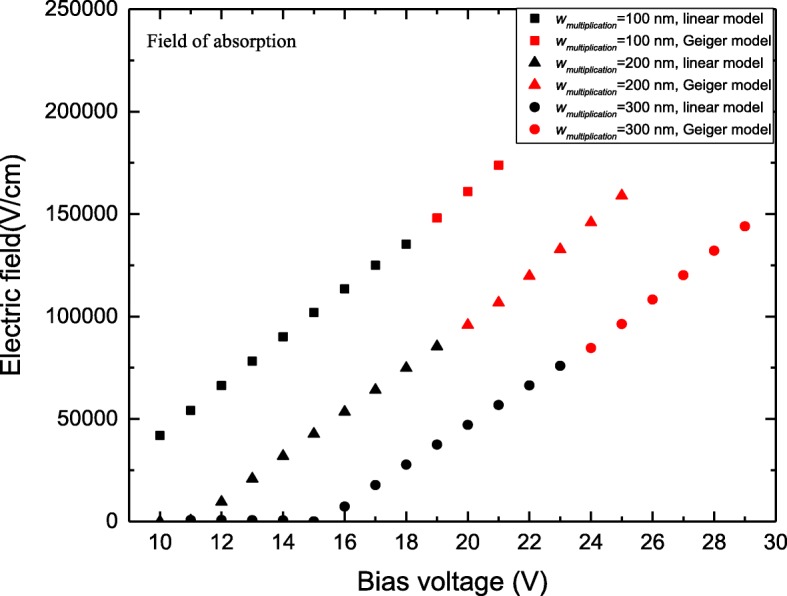
Simulation results electric field in absorption under the Geiger mode. The values of *W*_multiplication_ is 100 nm (black square), 200 nm (black triangle), 300 nm (black circle). Figure 4 presents the simulated field-voltage characteristics in the absorption layers under the Geiger mode. The thickness of the charge layer is 50 nm, and the thicknesses of the multiplication layer are 100, 200, and 300 nm, respectively.

Figures 4 and 5 present the simulated field-voltage characteristics in the multiplication and absorption layers under the Geiger mode, respectively. APDs operated in Geiger mode as SPADs are applied with a reverse bias that exceeds the breakdown voltage 1~6 V in the simulation. The thickness of the charge layer (*W*_charge_) is 50 nm, and the thicknesses of the multiplication layer (*W*_multiplication_) are 100, 200, and 300 nm, respectively.

When the InGaAs/InAlAs SPADs are operated under the linear model (APDs), the electric field in the absorption layer and multiplication layer increases linearly with increased bias voltage. However, as bias voltage exceeds the breakdown voltage under the Geiger mode, the electric field in the absorption layer increases linearly as before, whereas the increase in the avalanche electrical field in the multiplication layer becomes slow. Compared with InGaAs/InAlAs APDs operating in linear mode, the InGaAs/InAlAs SPADs achieve a high gain in the multiplication layer with the higher avalanche field, and a single photon can trigger a macroscopic current pulse. Concurrently, the field of absorption under the Geiger mode is larger than that under the linear model. Tunneling current becomes the dominant component of the dark current in the high field and a single photon can trigger a macroscopic current pulse with the avalanche gain, which is much larger than the linear mode.

### Design Consideration of SPADs

We know SAPDs work in a saturated mode. To maintain the high gain and small dark current, the electrical field control in the multiplication and absorption layers is important. If the field in absorption is less than the tunneling threshold field, it can maintain a high avalanche electrical field in the multiplication layer and avoid a tunneling current. Consequently, the concentration and the thickness of each layers should properly design for SPADs.

**Fig. 6:**
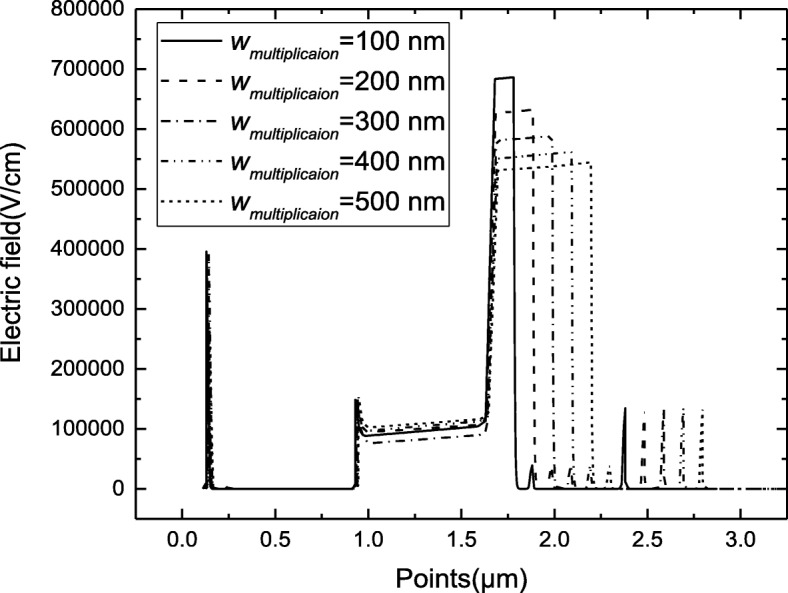
Electrical field in the multiplication layer with different *W*_multiplication_. Figure 5 presents the simulated field-voltage characteristics for different multiplication thicknesses (100–500 nm) when the device undergoes avalanche breakdown

Figure 2 shows that the SPADs have a probability of large tunneling effect because of the high field in the multiplication and absorption layers, which exceed the tunneling threshold electric field. Thus, the electric fields should be adjusted to lower than the tunneling threshold value both in InGaAs absorption and InAlAs multiplication. The theoretical analysis shows that the avalanche electrical field of multiplication is decreased by the products of *N*_charge_ and *w*_charge_ [28]. Thus, charge layer can control the field in absorption; however, the avalanche electrical field of the multiplication layer is determined by *w*_multiplication_. Figure 6 presents the simulated field-voltage characteristics for different multiplication thicknesses (100–500 nm) when the device undergoes avalanche breakdown. The background doping in the multiplication layer and absorption layer is 2 × 10^15^ cm^−3^, which is the intrinsic concentration of molecular beam epitaxy (MBE). The simulation results show that the avalanche electric field in the multiplication layer decreases with increased thickness of the multiplication layer. Thus, a thick multiplication layer can avoid the probability of tunneling effect through a low avalanche electrical field in multiplication.

To avoid the avalanche electrical field in multiplication exceeding the tunneling threshold value under the Geiger mode, the thickness of multiplication should be > 300 nm, which has an avalanche electrical field lower than 6 × 10^5^ V/cm and even exceeds the breakdown voltage in Fig. 4. Thus, a thick multiplication layer can avoid the tunneling effect in SPADs that under the Geiger mode. It is the reason that low DCR in SPADs with a thick multiplication.

**Fig. 7:**
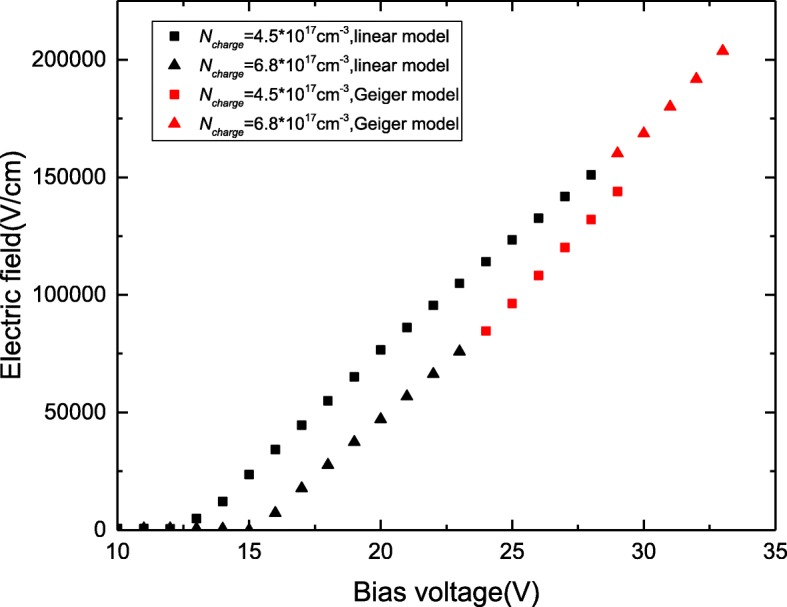
Field in the absorption layer with different *N*_charge_. The values of *N*_charge_ is 4.5*10^17^ cm^− 3^ (black square), 6.8*10^17^ cm^−3^ (black triangle). Figure 6 presents the electric-field distribution of absorption for different doping concentrations in the charge layer (*W*_charge_ = 50 nm)

As mentioned in section A, the electric field in the absorption layer increases linearly under the Geiger mode. The increase in bias voltage significantly influences the electric field in the absorption layer, which induces the field to have a large probability exceeding 2.0 × 10^5^ V/cm. Figure 7 presents the simulated electric-field distribution for different doping concentrations in the charge layer (*w*_charge_ = 50 nm). We find that higher doping concentrations have a low electric field in absorption layer and even exceeds the breakdown voltage of 5 V under the Geiger mode; however, at lower doping concentrations, the tunneling threshold electric field is quickly achieved. Consequently, the smaller doping concentrations in the charge layer cause earlier tunneling-effects initiation. To acquire sufficient operating bias voltage under the Geiger mode, the *N*_charge_ of SPADs is larger than the *N*_charge_ of APDs. Compared with the lower *N*_charge_ of SPADs, the higher *N*_charge_ of SPADs can work under a large bias voltage to avoid the tunneling effect and achieve high gain in the multiplication layer.

## Conclusions

We study the electric-field distribution and tunneling effect of InGaAs/InAlAs APDs and SPADs by theoretical analysis and simulation. When the InGaAs/InAlAs SPADs are operated under the Geiger mode, the electric field in the absorption layer increases linearly and deviates down from its linear relations. Considering the tunneling threshold electric field in multiplication layer, the thickness of the multiplication layer should be larger than 300 nm. Moreover, SPADs can work under a large bias voltage to avoid tunneling in absorption layer with high doping concentrations in the charge layer.

## Abbreviations

2D: Two-dimensional
APD: Avalanche photodiode
DCR: Dark count rate
SAGCMAPDs: Separate absorption, grading, charge, and multiplication avalanche photodiodes
SPAD: Single-photon avalanche photodiode
SPDE: Single-photon detection efficiency
